# Navigating a Surgical Dilemma: Conservative Management of a Rare Case of Spontaneous Duodenal Perforation in a Resource-Limited Setting

**DOI:** 10.7759/cureus.93098

**Published:** 2025-09-24

**Authors:** Yatawathurage Dananjaya M Jayaprasad, Arunajith G Peiris, Sandun Kularathna, Thamindu Madushan

**Affiliations:** 1 Surgery, Postgraduate Institute of Medicine, Colombo, LKA; 2 Surgery, District General Hospital, Horana, LKA

**Keywords:** conservative management, contrast-enhanced computed tomography, helicobacter pylori, multidisciplinary care, non-traumatic duodenal perforation, nsaids, peptic ulcer disease, peptic ulcer disease (pud), pneumoperitoneum, resource-limited setting

## Abstract

Non-traumatic duodenal perforation is a rare and potentially life-threatening condition, typically caused by peptic ulcer disease, and traditionally requires urgent surgical intervention. However, in resource-limited settings, conservative management can be an effective alternative for selected patients, particularly those with localized contamination and comorbidities that make surgery high-risk. This case report describes the successful non-operative management of a 78-year-old woman with poorly controlled type 2 diabetes, hypertension, dyslipidemia, and bronchial asthma, who presented with a seven-day history of right upper quadrant pain. Imaging with contrast-enhanced computed tomography revealed a localized second-part duodenal perforation with perihepatic extravasation of contrast, but no diffuse peritonitis. Given her frailty, a non-surgical approach was adopted, involving intravenous fluids, broad-spectrum antibiotics, proton pump inhibitors, and careful monitoring. The patient’s condition improved progressively, with pain resolution and return to a light diet by day 10, and she was discharged on day 11. This case underscores the importance of precise imaging, judicious patient selection, and adaptive resource utilization in managing duodenal perforation in settings with limited resources, highlighting the feasibility of conservative management in high-risk patients.

## Introduction

Non-traumatic perforation of the duodenum represents a rare but potentially life-threatening condition, with mortality reaching up to 25% in published studies [1].

Peptic ulcer disease secondary to Helicobacter pylori infection and/or non-steroidal anti-inflammatory drugs (NSAIDs) is currently the leading cause of non-traumatic duodenal perforation [1]. It can also occur in people with conditions such as duodenal diverticula [2], autoimmune conditions such as Crohn’s disease [3], and rarely tumors [4] and impacted gallstones in the duodenum [5].

Traditional management mandates urgent surgical exploration, particularly in the elderly (>70 years) and in patients with a delayed presentation [6]. However, there is clinical evidence supporting conservative strategies in hemodynamically stable patients with localized contamination, particularly those deemed high-risk for surgery [6,7]. The rationale behind the non-operative management (NOM) of duodenal perforation is that, in the case of small localized perforations, the ulcer seals by adhesion to surrounding structures, particularly to the posterior aspect of the liver quadrate lobe. Based on clinical observations and imaging studies, approximately 50% of perforated duodenal ulcers spontaneously sealed before surgical intervention [7]. Conservative strategies in the management of duodenal perforations include intravenous hydration with strict Nil-per-oral status, nasogastric decompression, proton pump inhibitors, and intravenous antibiotics. Finally, a follow-up endoscopy needs to be planned at 4-6 weeks [6].

NOM of spontaneous duodenal perforation is not without risk, as it is associated with dangers such as persistent leakage, intra-abdominal abscesses, and sepsis [8]. Furthermore, while the concept of NOM is established, there is a notable absence of detailed reports on its successful application in resource-limited environments. This gap in the literature is particularly relevant given the critical challenges in such settings, where surgical resources, specialized care, and advanced supportive therapies may be limited.

This case report highlights the effective conservative treatment of a non-traumatic duodenal perforation in a 78-year-old woman with multiple comorbidities, underscoring the essential importance of imaging, collaborative care from various disciplines, and judicious resource utilization in resource-constrained settings.

## Case presentation

A 78-year-old female with poorly controlled type 2 diabetes mellitus, hypertension, dyslipidemia, and bronchial asthma presented with a seven-day history of progressive right upper quadrant (RUQ) abdominal pain. The pain, initially mild, intensified acutely before admission and was said to get worse postprandially. She denied fever, vomiting, melena, or trauma. She had not been previously diagnosed or treated for peptic ulcer disease, and there was no history of NSAID abuse, past abdominal surgery, or upper gastrointestinal endoscopy.

Physical examination revealed localized RUQ tenderness and guarding, alongside tachycardia (110 bpm) but normotension. Additional findings included a temperature of 37.6 °C, a respiratory rate of 22/min, and oxygen saturation of 98% on room air. The abdomen was soft elsewhere, with no rebound tenderness, rigidity, or diffuse peritonitis.

**Figure 1:**
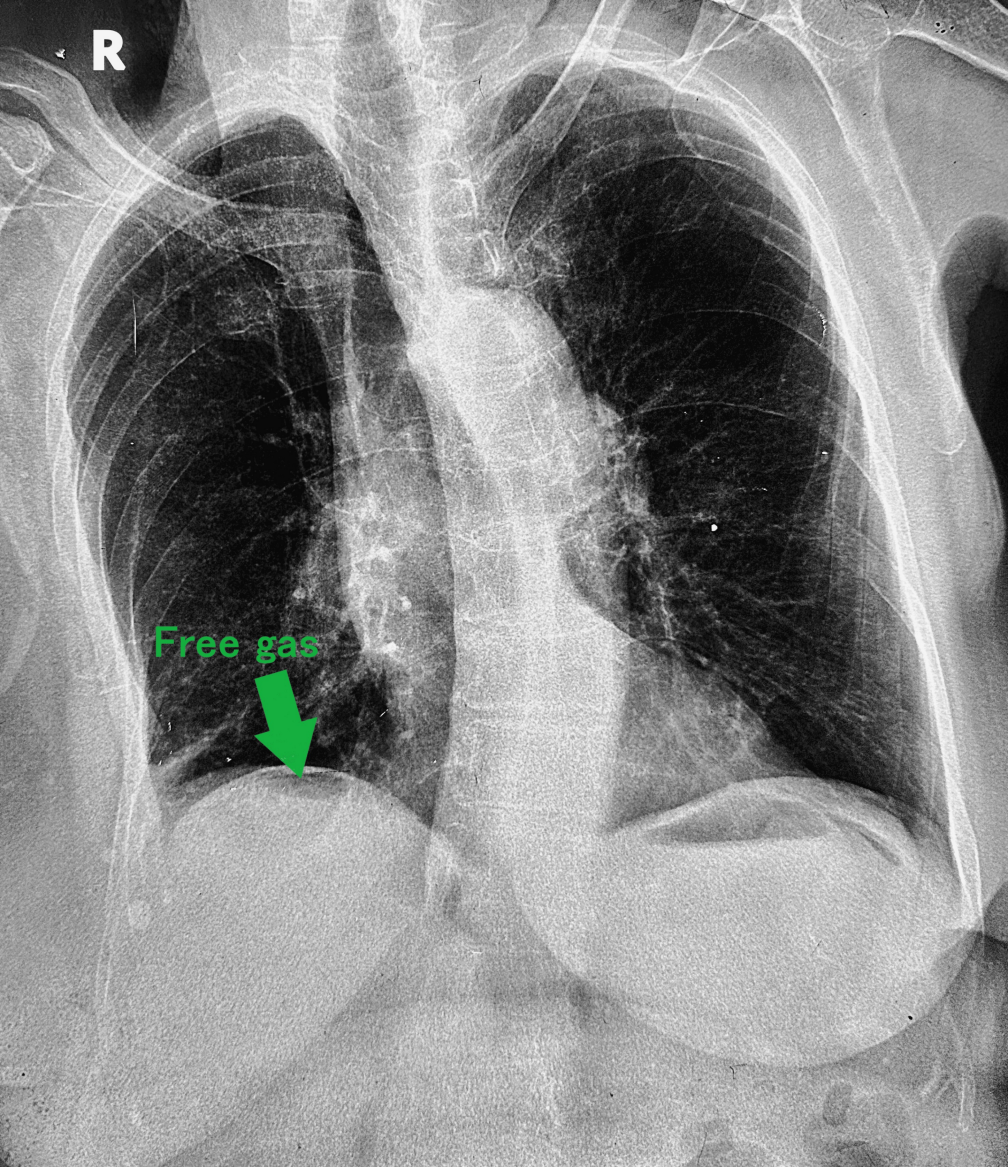
Erect chest X-ray demonstrating pneumoperitoneum. Erect chest X-ray showing subdiaphragmatic free air (free gas, marked by an arrow), consistent with pneumoperitoneum. This finding suggests the presence of intra-abdominal perforation, necessitating urgent clinical evaluation.

**Figure 2:**
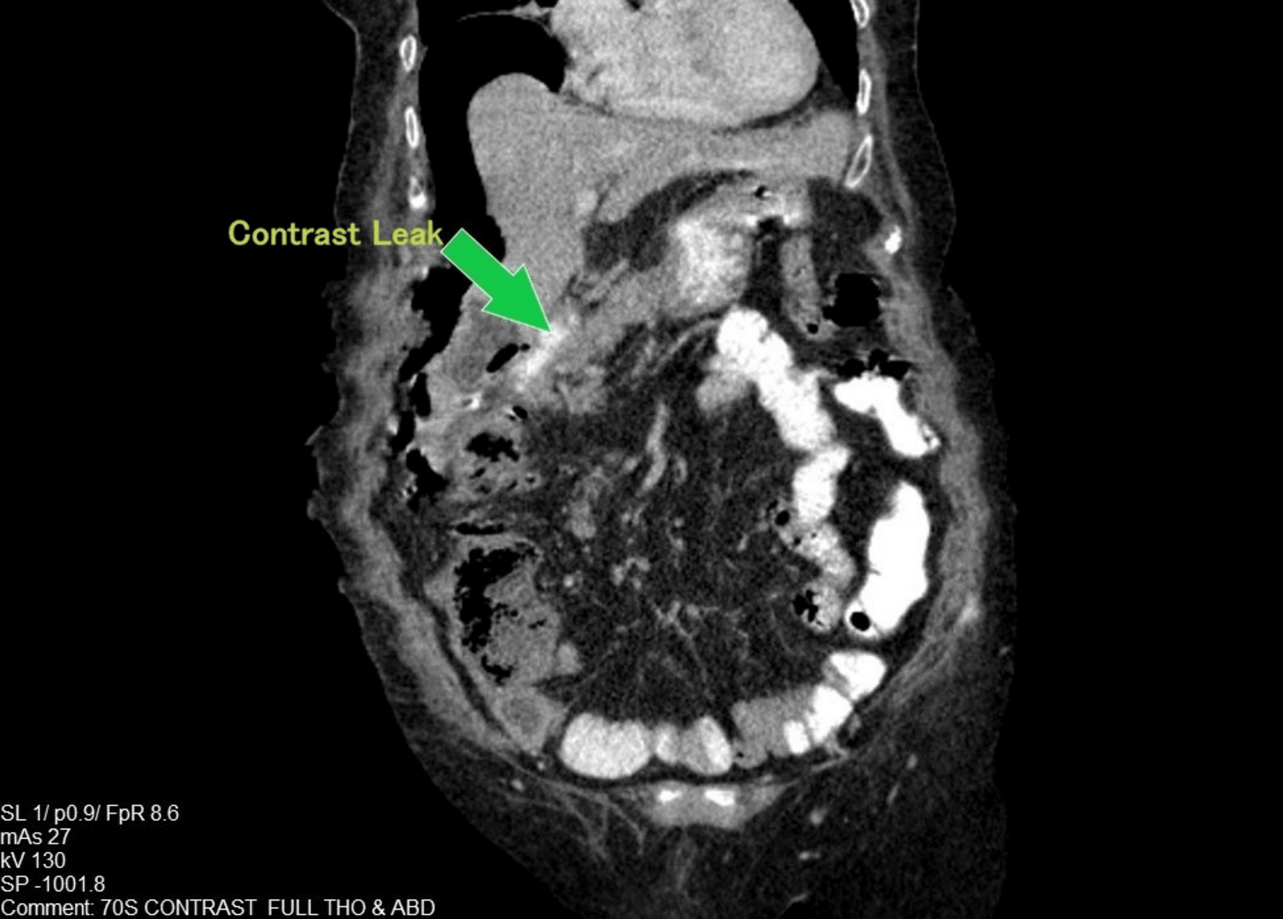
Coronal CECT demonstrating the site of duodenal perforation. Coronal view of a contrast-enhanced CT (CECT) scan showing oral contrast extravasation (contrast leak, marked by an arrow) from the second part of the duodenum. This radiological finding confirms the perforation and correlates with the free gas seen on the erect chest X-ray. Image credit: Department of Radiology, District General Hospital, Horana.

Laboratory findings included leukocytosis (25,000/uL, 85% neutrophils), elevated C-reactive protein (51 mg/L), and elevated serum creatinine of 1.9 mg/dL. Serum amylase, liver enzymes, and electrolytes were unremarkable. Erect chest radiography demonstrated subdiaphragmatic free air, indicating pneumoperitoneum (Figure 1). Abdominal ultrasound did not reveal intra-abdominal collections and failed to identify the cause of the pneumoperitoneum. Subsequent oral contrast-enhanced computed tomography (CT) localized the perforation to the second part of the duodenum, with contrast extravasation confined to the right perihepatic space (Figure 2). No diffuse peritonitis or distal intraperitoneal spillage was observed.

The main differential diagnoses at presentation included acute cholecystitis, perforated gastric ulcer, and acute pancreatitis. Acute cholecystitis was excluded based on the absence of gallbladder pathology on ultrasound, while acute pancreatitis was ruled out due to a normal serum amylase level and the lack of suggestive findings on imaging. CT findings of localized contrast extravasation at the second part of the duodenum ultimately confirmed the diagnosis of duodenal perforation in a hemodynamically stable patient.

Treatment

Given the patient’s frailty, comorbidities, hemodynamic stability and localized contamination on CT, a non-operative approach was prioritized. Management included strict nil-per-oral status and intravenous hydration with normal saline and 5% dextrose administered alternatively. This regimen aimed to maintain electrolyte balance, prevent starvation ketosis through glucose provision, and mitigate hyperchloremic acidosis [9]. Hourly fluid requirements were calculated based on the patient’s weight (45 kg) and adjusted according to urine output (>0.5 mL/kg/hour) and hemodynamic parameters. Careful fluid status assessments were conducted despite resource limitations in a high-volume ward setting. Broad-spectrum antibiotics (Co-amoxiclav 1.2 g intravenously (IV) every 8 hours, Metronidazole 500 mg IV every 8 hours) were initiated for enteric flora coverage, alongside proton pump inhibition (omeprazole 40 mg IV twice daily) to reduce gastric acidity. Despite plans for nasogastric decompression and total parenteral nutrition (TPN), the patient declined the former, and institutional limitations precluded the latter. Protein requirements were addressed via intermittent fresh frozen plasma transfusions.

A structured monitoring plan was implemented, including 6-hourly clinical reviews, serial abdominal examinations, and daily blood investigations (Full blood Count, C-reactive protein, Serum creatinine, and electrolytes). Surgery was to be reconsidered if the patient developed diffuse peritonitis, persistent sepsis, or hemodynamic instability [8]. Follow-up imaging on day 5 (abdominal X-ray and ultrasound) demonstrated reduced free air and no new intra-abdominal collections, supporting continued conservative management. A repeat CT scan was not performed due to resource limitations and progressive clinical improvement. Laboratory values normalized over the course of treatment, with leukocytosis resolving by day 7 (WBC 9,500/µL) and creatinine stabilizing at 0.8 mg/dL by day 5.

The patient was kept nil by mouth for a total of 6 days. By the seventh day, abdominal pain and tenderness significantly reduced, permitting the initiation of sips of clear fluids. Over the subsequent days, oral liquid intake gradually advanced, and by day 10, she tolerated a light diet consisting of jelly, yogurts, eggs, and milk rice.

Outcome and follow-up

By day 11, the patient was pain-free with a soft, non-tender abdomen. She was transferred to the medical ward for management of right lower lobe pneumonia and discharged with outpatient surgical follow-up. Upper GI endoscopy was deferred during the acute phase due to risks of exacerbating the perforation; instead, it was scheduled for six weeks post-discharge to exclude malignancy [8]. Upon discharge, long-term proton pump inhibitors were prescribed, and empirical Helicobacter pylori eradication therapy was initiated with a two-week regimen, as diagnostic testing was not available in our setting. We acknowledge that empirical eradication is not ideal, and the recommended approach is a test-and-treat strategy; however, resource limitations necessitated this pragmatic approach in our patient [10].

## Discussion

This case highlights three pivotal themes in the conservative management of non-traumatic duodenal perforation: *diagnostic challenges*, *risk stratification*, and *adaptive resource utilization*.

First, the retroperitoneal nature of duodenal perforations often masks classical peritonitis signs, as seen here, where guarding was localized rather than diffuse. Contrast-enhanced CT proved indispensable by demonstrating confined perihepatic leakage. An abdominal contrast-enhanced CT scan has become the preferred imaging technique due to its high sensitivity, which is reportedly 98% [11].

Second, conservative management hinges on strict patient selection. Hemodynamic stability, absence of diffuse peritonitis, and controlled sepsis are prerequisites, as outlined in the World Society of Emergency Surgery (WSES) 2020 guidelines [6]. Our patient's advanced age and cardiopulmonary comorbidities rendered surgery prohibitively risky, aligning with Thorsen et al. [12], who reported a 40% mortality rate in elderly patients undergoing emergency laparotomy for perforated peptic ulcers.

Third, resource limitations necessitated pragmatic adaptations. While TPN remains the gold standard for prolonged nil-per-os states, institutional unavailability compelled reliance on fresh frozen plasma (FFP) transfusions to address hypoalbuminemia and protein-calorie deficits. While specific reports on the use of FFP supplementation in place of TPN for managing hypoalbuminemia in duodenal perforation cases are scarce, the principle of adapting treatment strategies to available resources is a recognized practice in low-resource settings. It is important to note that the use of FFP as a nutritional supplement is not standard practice [13]. Decisions should be guided by clinical judgment, weighing the potential benefits against risks, and tailored to the individual patient's condition and the specific resource constraints of the healthcare setting.

## Conclusions

This case demonstrates that conservative management of non-traumatic duodenal perforation is feasible in carefully selected patients, provided that precise imaging, vigilant monitoring, and multidisciplinary collaboration are ensured. Our findings reinforce the WSES emphasis on individualized approaches to gastrointestinal emergencies, particularly in elderly, high-risk cohorts and in resource-limited settings. Importantly, this is a single case, and the report is not intended to change standard practice but rather to illustrate that cautious patient selection and close observation may allow non-operative management in exceptional circumstances. By sharing this experience, we hope to contribute practical insights that may inform clinical decision-making when surgery is deemed prohibitively risky or unavailable. Future research should focus on developing standardized protocols for conservative treatment, including pragmatic nutritional support strategies tailored for low-resource environments.
